# *Staphylococcus aureus sarA* Regulates Inflammation and Colonization during Central Nervous System Biofilm Formation

**DOI:** 10.1371/journal.pone.0084089

**Published:** 2013-12-30

**Authors:** Jessica N. Snowden, Matt Beaver, Karen Beenken, Mark Smeltzer, Alexander R. Horswill, Tammy Kielian

**Affiliations:** 1 Department of Pediatrics, University of Nebraska Medical Center, Omaha, Nebraska, United States of America; 2 Department of Pathology and Microbiology, University of Nebraska Medical Center, Omaha, Nebraska, United States of America; 3 Department of Microbiology and Immunology, University for Arkansas for Medical Sciences, Little Rock, Arkansas, United States of America; 4 Department of Microbiology, Carver College of Medicine, University of Iowa, Iowa City, Iowa, United States of America; National Institutes of Health, United States of America

## Abstract

Infection is a frequent and serious complication following the treatment of hydrocephalus with CSF shunts, with limited therapeutic options because of biofilm formation along the catheter surface. Here we evaluated the possibility that the *sarA* regulatory locus engenders *S. aureus* more resistant to immune recognition in the central nervous system (CNS) based on its reported ability to regulate biofilm formation. We utilized our established model of CNS catheter-associated infection, similar to CSF shunt infections seen in humans, to compare the kinetics of bacterial titers, cytokine production and inflammatory cell influx elicited by wild type *S. aureus* versus an isogenic *sarA* mutant. The *sarA* mutant was more rapidly cleared from infected catheters compared to its isogenic wild type strain. Consistent with this finding, several pro-inflammatory cytokines and chemokines, including IL-17, CXCL1, and IL-1β were significantly increased in the brain following infection with the *sarA* mutant versus wild type *S. aureus*, in agreement with the fact that the *sarA* mutant displayed impaired biofilm growth and favored a planktonic state. Neutrophil influx into the infected hemisphere was also increased in the animals infected with the *sarA* mutant compared to wild type bacteria. These changes were not attributable to extracellular protease activity, which is increased in the context of SarA mutation, since similar responses were observed between *sarA* and a *sarA/*protease mutant. Overall, these results demonstrate that *sarA* plays an important role in attenuating the inflammatory response during staphylococcal biofilm infection in the CNS via a mechanism that remains to be determined.

## Introduction

Ventricular shunts have resulted in drastic improvements in survival and neurologic outcomes in children with hydrocephalus [1]. However, these shunts can be associated with significant complications, including infection, reported in 3–20% of children with catheters in the central nervous system (CNS; [1]). These infections have been associated with increased mortality rates, increase seizure risk and IQ loss [2]. *Staphylococcus aureus* (*S. aureus*) and *Staphylococcus epidermidis* are the most common causes of CNS shunt infections and both organisms are well known to form biofilms on catheter surfaces [2], [3]. Biofilms are classically resistant to antibiotic therapy, such that catheter removal is currently recommended for the treatment of these infections [4].

Very few animal models have been described that investigate the pathogenesis of biofilm infections within the CNS. Our laboratory has previously described a mouse model of *S. aureus* catheter-associated biofilm infection, which mimics several aspects of ventricular shunt infections in humans [5]. This model is typified by innate immune cell influx into the tissue surrounding the infected catheter, with a concomitant increase in inflammatory cytokine and chemokine expression compared to sterile catheter placement [5]. This was similar to the inflammatory phenotype observed in studies utilizing a steel pin tibial biofilm infection model [6], but in contrast to the anti-inflammatory immune response reported in a flank catheter biofilm model, emphasizing the variability in immune responses to biofilm infection in different body compartments [7], [8], [9]. To better elucidate the impact of biofilm formation on the inflammatory response within the CNS, we compared our catheter-associated biofilm infection with a parenchymal brain abscess utilizing the methicillin-susceptible *S. aureus* (MSSA) strain ACH 1719. Using the same bacterial strain, but under different growth modalities, would better define the inflammatory impact of biofilm versus planktonic infection in the CNS compartment.

The staphylococcal accessory regulatory (*sarA*) locus encodes a DNA-binding protein (SarA) that has a global impact on gene expression in *S. aureus* [10]–[12]. Several studies have demonstrated that mutation of the *sarA* locus limits, but does not abolish, the ability of most *S. aureus* strains to form a biofilm and results in greater susceptibility to anti-staphylococcal antibiotic treatment [10]–[12]. With decreased biofilm formation, there is likely a greater degree of planktonic growth in infected tissues, which would provide both antibiotics and the immune response increased access to bacteria compared to a fully intact biofilm. Indeed, we found in the current study that when compared with both parenchymal brain abscesses and a *sarA* mutant, which displayed impaired biofilm formation in the CNS, catheter-associated biofilm infections established with wild type *S. aureus* were significantly less inflammatory. Specifically, both cytokine and chemokine levels in the tissues surrounding infected catheters as well as innate immune cell influx were significantly decreased in the brains of mice infected with wild type *S. aureus* that formed a mature biofilm [5]. These findings were consistent with our prediction that biofilm growth actively attenuates the CNS host immune response in favor of bacterial persistence. This builds on findings from our earlier work, showing inflammation during CNS catheter infection in comparison to sterile catheters [5], by demonstrating that this inflammation is due to the parenchymal spread observed during infection rather than the biofilm. The enhanced CNS inflammatory response observed with the *sarA* mutant was protease-independent, since similar inflammatory profiles were elicited with the *sarA* and a *sarA*/protease mutant [13]. These studies highlight the role of the *sarA* regulatory locus, which may engender *S. aureus* more resistant to CNS immunity based on its ability to regulate biofilm formation via an undetermined mechanism. Understanding the role of *sarA* in CNS catheter infections may facilitate the identification of viable therapeutic targets for the treatment of ventricular shunt infections.

## Results

### *S. aureus* Catheter-associated Biofilms are Less Inflammatory than Parenchymal Brain Infection in the CNS

To delineate potential alterations in the CNS immune response that occur based on infection modality alone, CNS catheter-associated infection was compared with a parenchymal brain abscess using the same *S. aureus* strain. As previously reported, our CNS catheter infection model generates a *bona fide* biofilm, using a methicillin-susceptible *S. aureus* (MSSA) clinical strain originally isolated from a child with a ventriculoperitoneal shunt infection [5]. This infection elicits pro-inflammatory cytokine and chemokine release in the brain tissue surrounding infected compared to sterile catheters, although the relative contribution of bacteria adherent to the catheter versus detached organisms to this inflammatory response was unclear. Thus, we compared the intensity of immune responses generated following catheter-associated biofilm infection to a parenchymal brain abscess using the same MSSA isolate to allow direct comparisons of the impact of the infection modality on the inflammatory response within the CNS.

**Figure 1:**
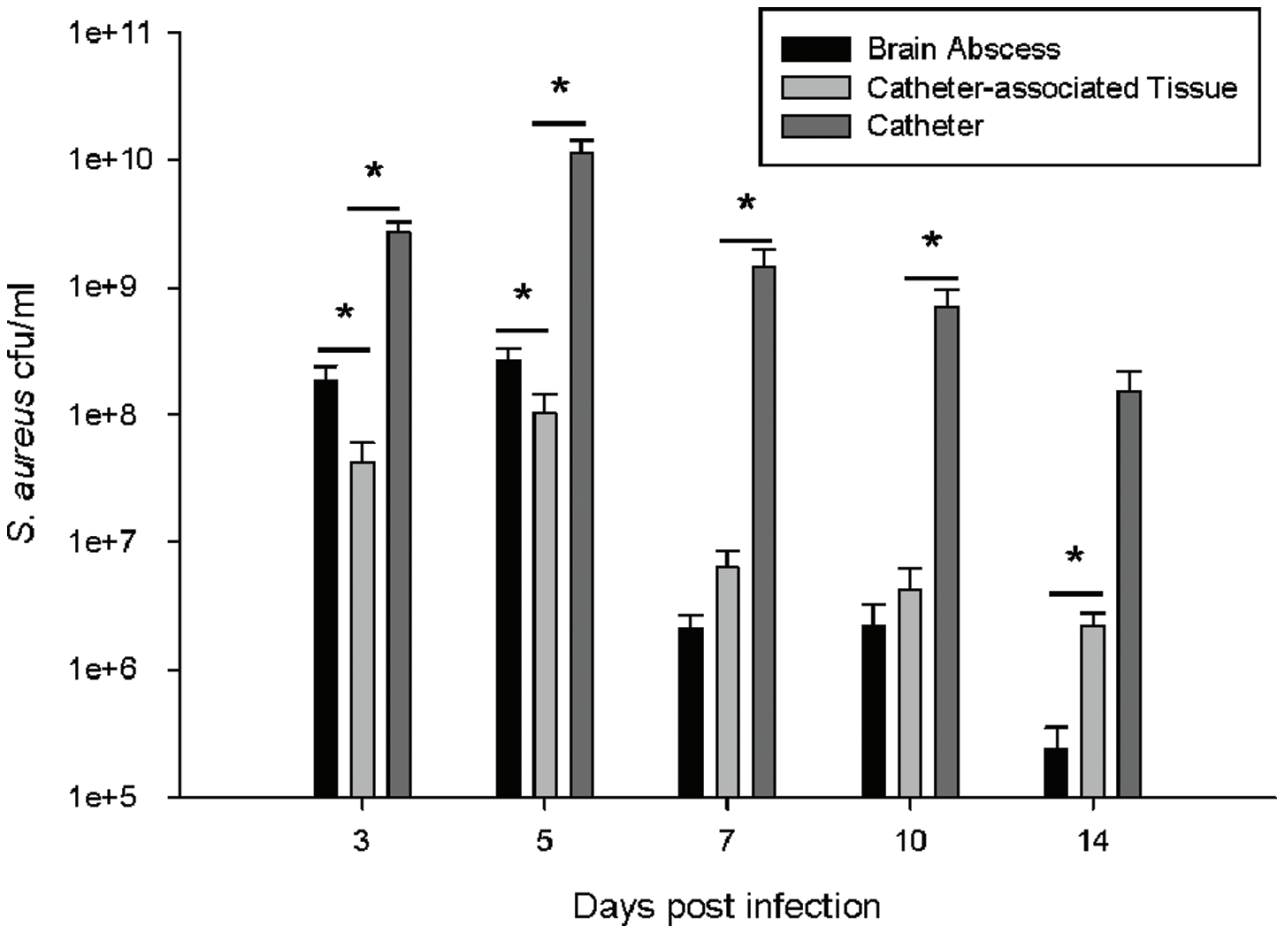
MSSA CNS catheter-associated biofilm infection persists longer than a parenchymal brain abscess. The infected tissue was removed, homogenized and cultured to enumerate bacterial burdens in the tissue containing the brain abscess or surrounding the infected catheter. Catheters were also removed, rinsed and sonicated for quantification of viable bacteria. * = p<0.05 (n = 12–18 mice/group/time point).

As observed in brain abscesses generated using other *S. aureus* strains [14]–[16], parenchymal bacterial burdens were initially high and began to rapidly decline by the first week (Figure 1). In contrast, during catheter-associated infection, bacterial titers on the catheter surface remained elevated throughout the course of infection, reflective of more chronic biofilm formation (Figure 1). Similar to brain abscesses, bacterial burdens in the tissues surrounding infected catheters also progressively decreased over time, reflecting a planktonic growth state under both conditions.

**Figure 2:**
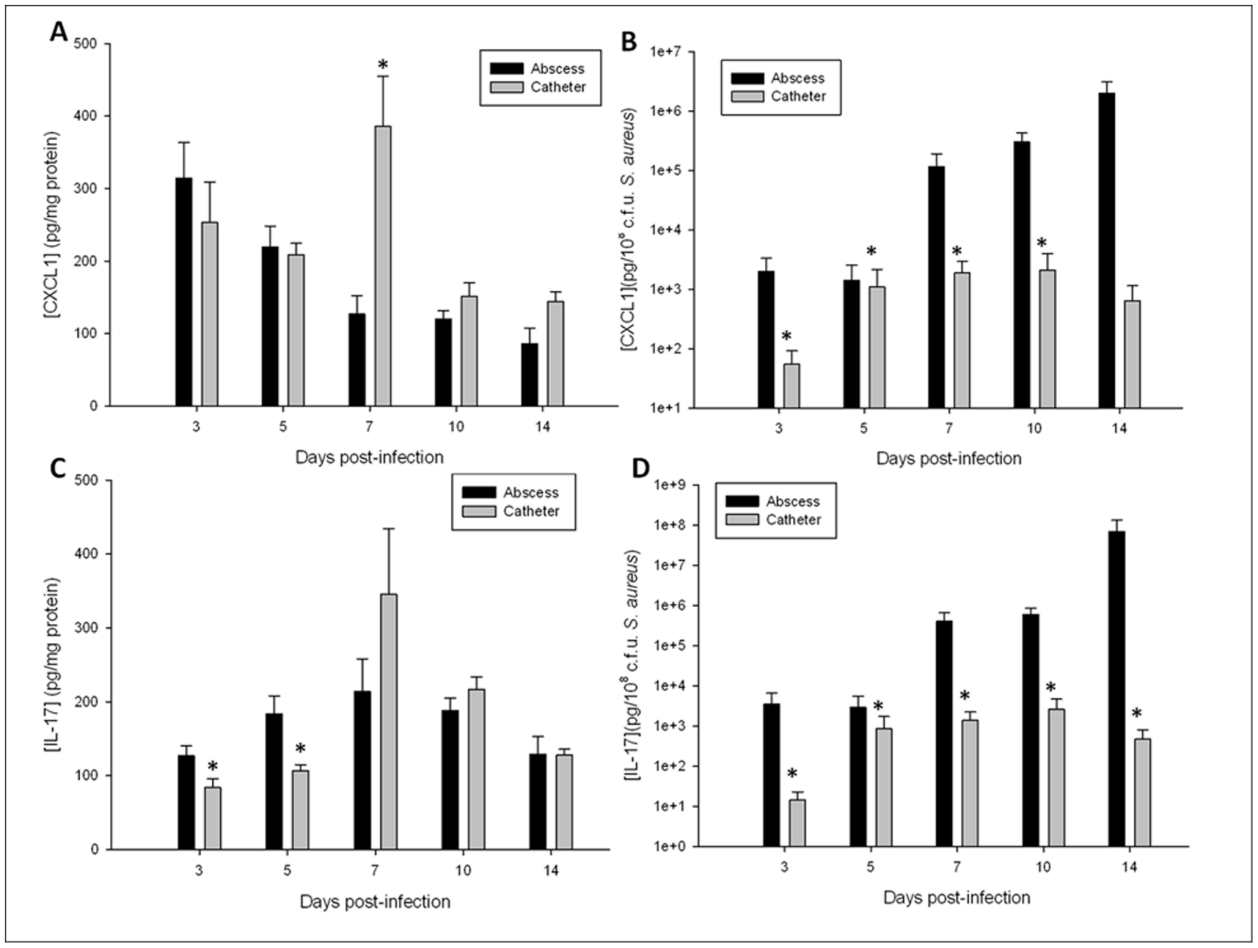
CNS catheter-associated biofilm infection is associated with attenuated inflammation compared to parenchymal abscesses. The tissues encompassing the brain abscess and the tissues surrounding the infected catheters were homogenized and the resulting supernatants analyzed for levels of the pro-inflammatory mediators CXCL1 (A, B) and IL-17 (C, D). Raw cytokine/chemokine levels are reported (A, C) as well as correction for disparate bacterial burdens (B, D) * = p<0.05 (n = 12–18 mice/group/time point).

Next, comparisons in proinflammatory cytokine and chemokine expression between the catheter biofilm and brain abscess infection models were assessed. Initial examination of raw cytokine/chemokine values revealed similar CXCL1 and IL-17 expression in both models (Figure 2A and C, respectively) throughout the course of infection despite the dramatic differences in total bacterial burdens (Figure 1). To evaluate the proportional impact of the total bacterial burden in either the planktonic or biofilm infection model on inflammatory mediator production, cytokine and chemokine levels were adjusted based on total bacterial titers. After this normalization, CXCL1 and IL-17 were significantly decreased during biofilm infection compared to planktonic abscesses (Figure 2B and D, respectively). A similar trend was observed with IL-1β, with significantly increased levels observed in brain abscess tissues versus catheter-associated biofilm infection (data not shown). Collectively, these findings suggest that biofilms elicit less inflammation than a planktonic infection within the CNS compartment using the MSSA strain examined here.

### *sarA* is Critical for CNS Catheter-associated Biofilm Formation

The staphylococcal accessory regulatory (*sarA*) locus encodes a DNA-binding protein (SarA) that has a global impact on gene expression in *S. aureus* [10]–[12]. Several studies have demonstrated that mutation of the *sarA* locus limits, but does not abolish, the ability of most *S. aureus* strains to form a biofilm and results in greater susceptibility to anti-staphylococcal antibiotic treatment [10]–[12]. Importantly, strain-dependent differences among *S. aureus* isolates that affect biofilm phenotypes have been reported [12], [17], [18], so assessing the ability of the *sarA* mutant in the ACH1719 MSSA background to form a biofilm was essential [12], [17], [18]. To evaluate biofilm formation, ACH1719Δ*sarA* was compared with its isogenic wild type strain ACH1719, as well as two other *S. aureus* strains known to be effective biofilm producers, using an *in vitro* microtiter plate assay as well as confocal microscopy analysis. As expected, both of these assays demonstrated decreased biofilm development in ACH1719 Δ*sarA* in comparison to wild type ACH1719 (Figure S1).

To determine whether genes regulated by the *sarA* operon were involved in the diminished immune response during catheter-associated biofilm infections (Figure 2), we compared the course of CNS catheter-associated biofilm infection using wild type ACH1719 and ACH1719Δ*sarA*. Mice were observed following the surgical procedure for weight loss, clinical indices of infection, seizure activity and mortality. Mice infected with wild type ACH1719 had less weight loss and lower mortality rates than animals infected with ACH1719Δ*sarA* (15.9% ±1.3% vs 22.2% ±1.5% p<0.006 and 23% ±4.9% vs 29.8% ±6% n.s., respectively). This is in contrast to prior studies using *sarA-*deficient *S. aureus* in other infection models, in which a *sarA* mutant was less virulent than the wild type strain [19], [20]. This difference in virulence emphasizes the importance of evaluating host-pathogen interactions in multiple tissue compartments, as areas such as the CNS may respond differently to infection than peripheral sites, although strain background differences cannot be discounted. Both groups exhibited similar frequencies in seizure activity during the second week post-infection, with spontaneous recovery to baseline following these episodes.

**Figure 3:**
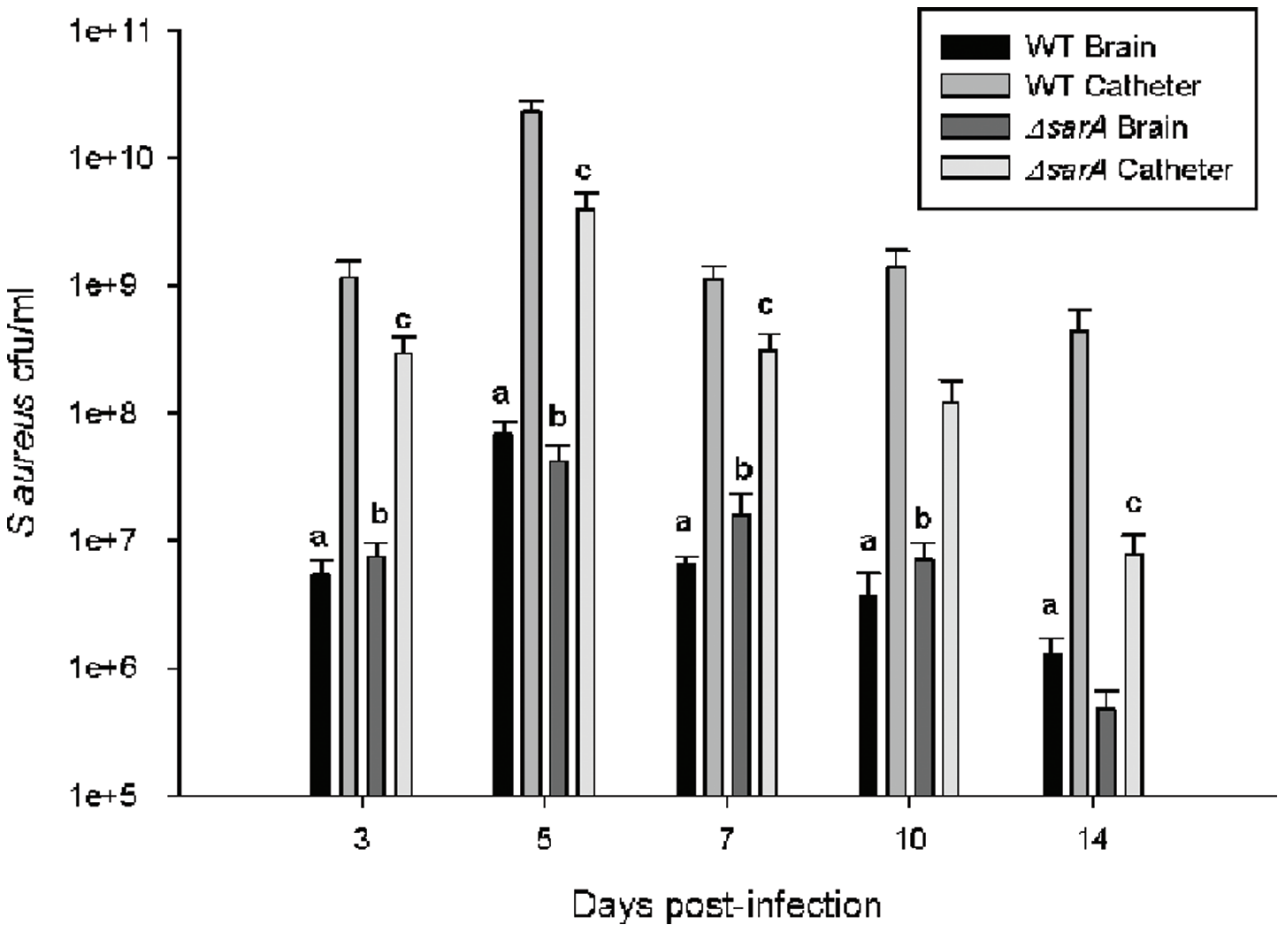
*sarA* is critical for catheter-associated biofilm formation in a CNS catheter infection model. Infected catheters were removed, rinsed and sonicated for quantification of viable bacteria associated with wild type ACH1719 (**WT**) or ACH1719Δ*sarA* (**Δ** ***sarA***) *S. aureus*. At all time points, bacterial burdens in brain parenchyma were lower than the corresponding catheter cultures (See Figure S2). * = p<0.05 WT catheter vs Δ*sarA* catheter; (n = 11–21 mice/group/time point).

As expected based on our *in vitro* studies, ACH1719 Δ*sarA* displayed impaired catheter-associated biofilm growth compared to its isogenic wild type strain (Figure 3). This diminished biofilm formation is consistent with observations in other animal models of *sarA-*deficient *S. aureus* infection [11], [19], [20] and our *in vitro* assays (Figure S1). Additionally, mice infected with ACH1719 Δ*sarA* demonstrated higher rates of bacterial clearance, with 43% (10/23) of animals having no discernible bacterial growth on catheters at day 14, versus 15% (2/13) of those infected with wild type ACH1719. There was no evidence of bacterial spread to either the contralateral brain hemisphere or peripheral organs in either wild type or ACH1719 Δ*sarA* (data not shown). Finally, there were no significant differences in bacterial burdens in the surrounding brain parenchyma between wild type and ACH1719 Δ*sarA* infected mice at any time point examined (Figure S2).

### *sarA* Mutation Leads to Heightened Proinflammatory Responses during CNS Catheter-associated Infection

**Figure 4:**
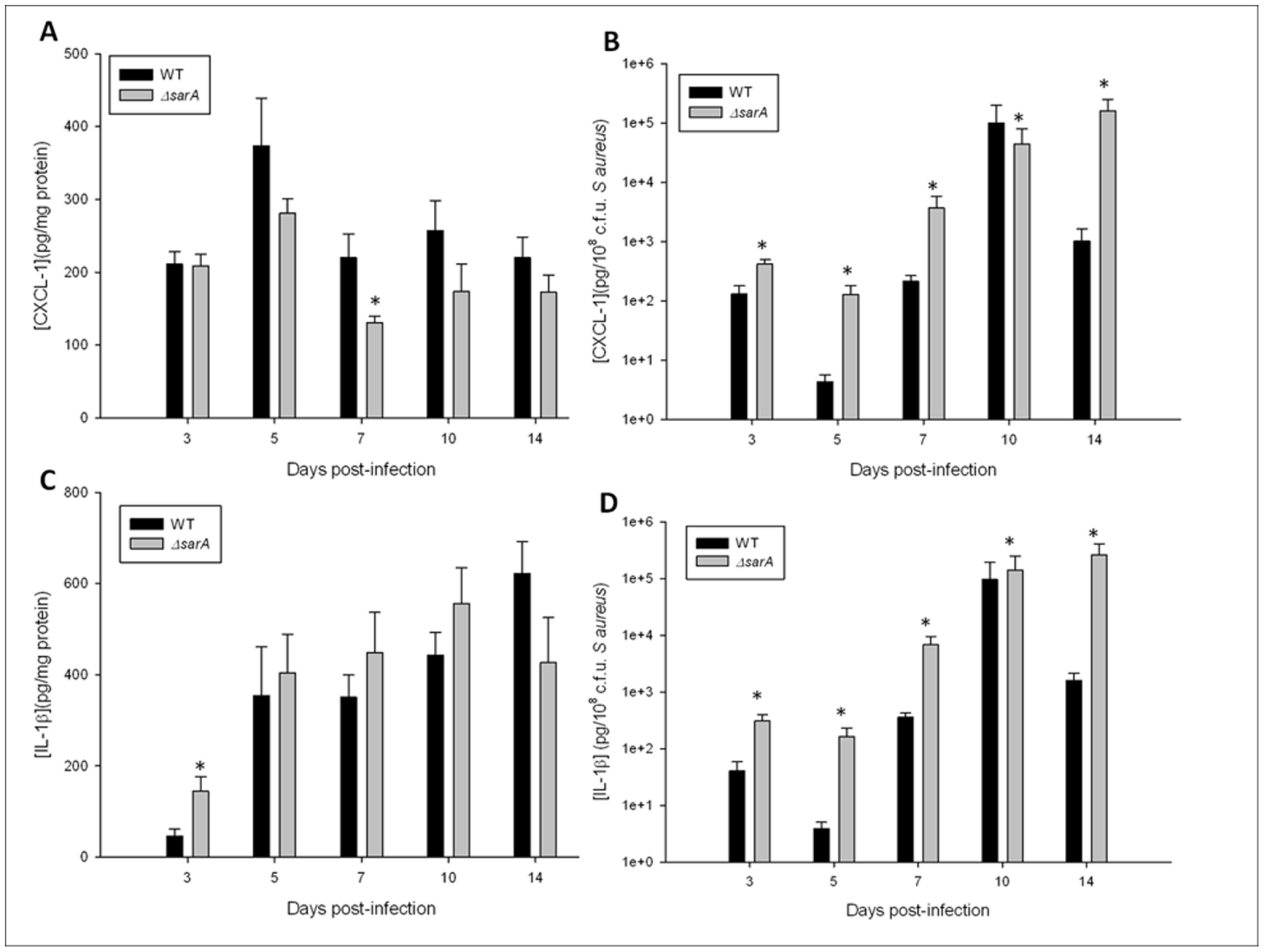
*sarA* dictates the extent of proinflammatory mediator expression during CNS catheter-associated infection. Catheter-associated infections were generated with either wild type ACH1719 (**WT**) or ACH1719Δ*sarA* (**Δ** ***sarA***). The tissue surrounding the infected catheters was homogenized and the resulting supernatant analyzed for levels of the pro-inflammatory mediators CXCL1 (A, B) and IL-1β (C, D). Results are presented as raw data (A and C) and after adjustment for divergent bacterial burdens (B and D). * = p<0.05 (n = 11–21 mice/group/time point).

To evaluate whether *sarA* expression, and indirectly biofilm growth, attenuates pro-inflammatory mediator production during CNS catheter infection, homogenates of catheter-associated tissues were analyzed for IL-1β and CXCL1 production by ELISA. When adjusted to account for the divergent bacterial burdens between the two strains, both mediators were significantly elevated in response to ACH1719 Δ*sarA* compared to WT bacteria (Figure 4). A similar pattern was seen with IL-17, with significantly elevated production during ACH1719 Δ*sarA* infection (data not shown). These findings are consistent with cytokine/chemokine analysis of catheter-associated versus parenchymal brain abscess infection (Figure 2), reinforcing the concept that the ability to form a biofilm is inherently anti-inflammatory in the CNS.

**Figure 5:**
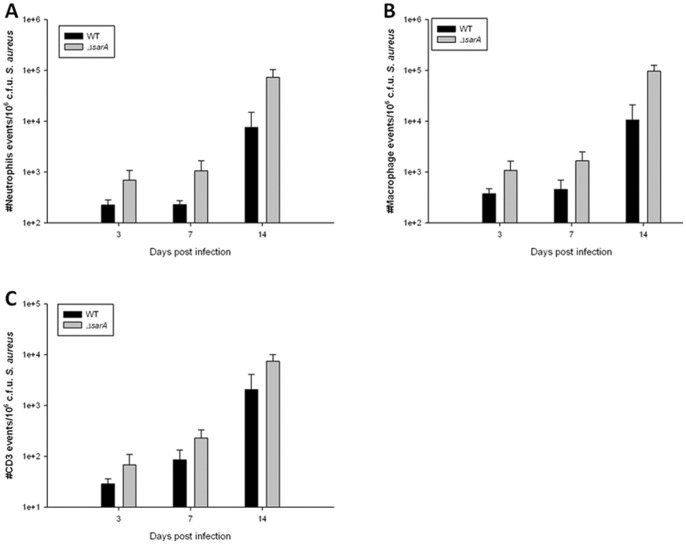
*sarA* influences peripheral immune cell influx during CNS catheter-associated infection. Catheter associated cells were recovered from mice at days 3, 7 and 14-infection, whereupon pooled tissue from 4–5 mice per group (wild type ACH1719– **WT** vs *ACH1719*Δ*sarA* – **Δ** ***sarA***) was analyzed for neutrophil (A), macrophage (B) and T cell (C) infiltrates, with three independent replicates. Data was corrected for bacterial burdens at each time point. See Figure S2 for representative histograms.

Given the elevated levels of pro-inflammatory cytokines associated with the ACH1719 Δ*sarA* strain, a concomitant increase in innate immune cells was expected. To quantitate innate immune cell influx, catheter-associated leukocytes were recovered using a Percoll gradient method and analyzed by FACS. A trend towards increased numbers of neutrophils, macrophages and T lymphocytes were observed following infection with the biofilm-deficient ACH1719 Δ*sarA* strain compared to wild *type S. aureus*, although these differences did not reach statistical significance (Figure 5, Figure S3), which correlated with enhanced chemokine expression (Figure 4).

### *sarA* Mutation Leads to Decreased α-toxin Production during CNS Catheter-Associated Infection

**Figure 6:**
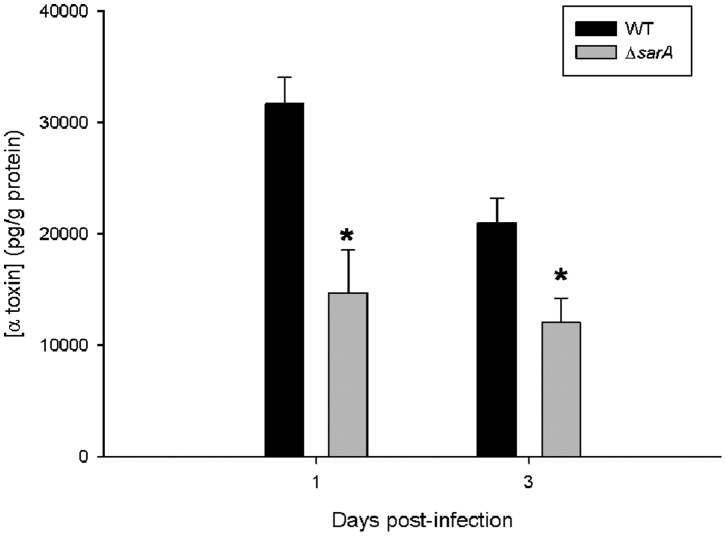
α-toxin levels are reduced during CNS catheter-associated infection with a *sarA* mutant. Alpha-toxin levels were measured in brain homogenates of animals infected with the ACH1719 *sarA* mutant (**Δ** ***sarA***) or wild type ACH1719 strain (**WT**) at the indicated time points post-infection using a custom-designed ELISA. * = p<0.05 (n = 13–15 mice/group/time point).

Prior studies have reported decreased α-toxin production by certain *S. aureus* USA300 *sarA* mutant strains *in vitro* and *in vivo* [13], [21], [22]. This is thought to result from increased production of extracellular proteases in *sarA-*deficient strains of staphylococci, leading to decreased accumulation of α-toxin as well as phenol soluble modulins (PSMs) [22]. However, other studies in laboratory-derived strains have demonstrated an opposite or no effect on α-toxin production in *S. aureus sarA* mutants [17], [22]. As α-toxin can be an inflammatory stimulus within the brain [23], [24], evaluating α-toxin production by this staphylococcal strain *in vivo* was assessed. As observed in other *S. aureus* clinical isolates, animals infected with ACH1719 Δ*sarA* demonstrated decreased levels of α-toxin at days 1 and 3 following infection (Figure 6). Later time points were not examined as bacterial burdens were declining in the tissue and α-toxin expression was expected to be below the threshold of detection based on other studies of staphylococcal infection in the brain (T Kielian, unpublished data). Given the pro-inflammatory nature of α-toxin, it was expected that inflammatory mediator expression would be higher in tissues associated with the wild type ACH1719 strain compared to ACH1719 Δ*sarA*, since the former displayed elevated α-toxin levels. However, the opposite relationship was observed (Figure 4), suggesting that the increased inflammation associated with the *sarA* mutant cannot be attributed to alterations in α-toxin levels in the surrounding tissue.

### The Heightened CNS Inflammatory Response Elicited by a *sarA* Mutant is Largely Protease-independent

**Figure 7:**
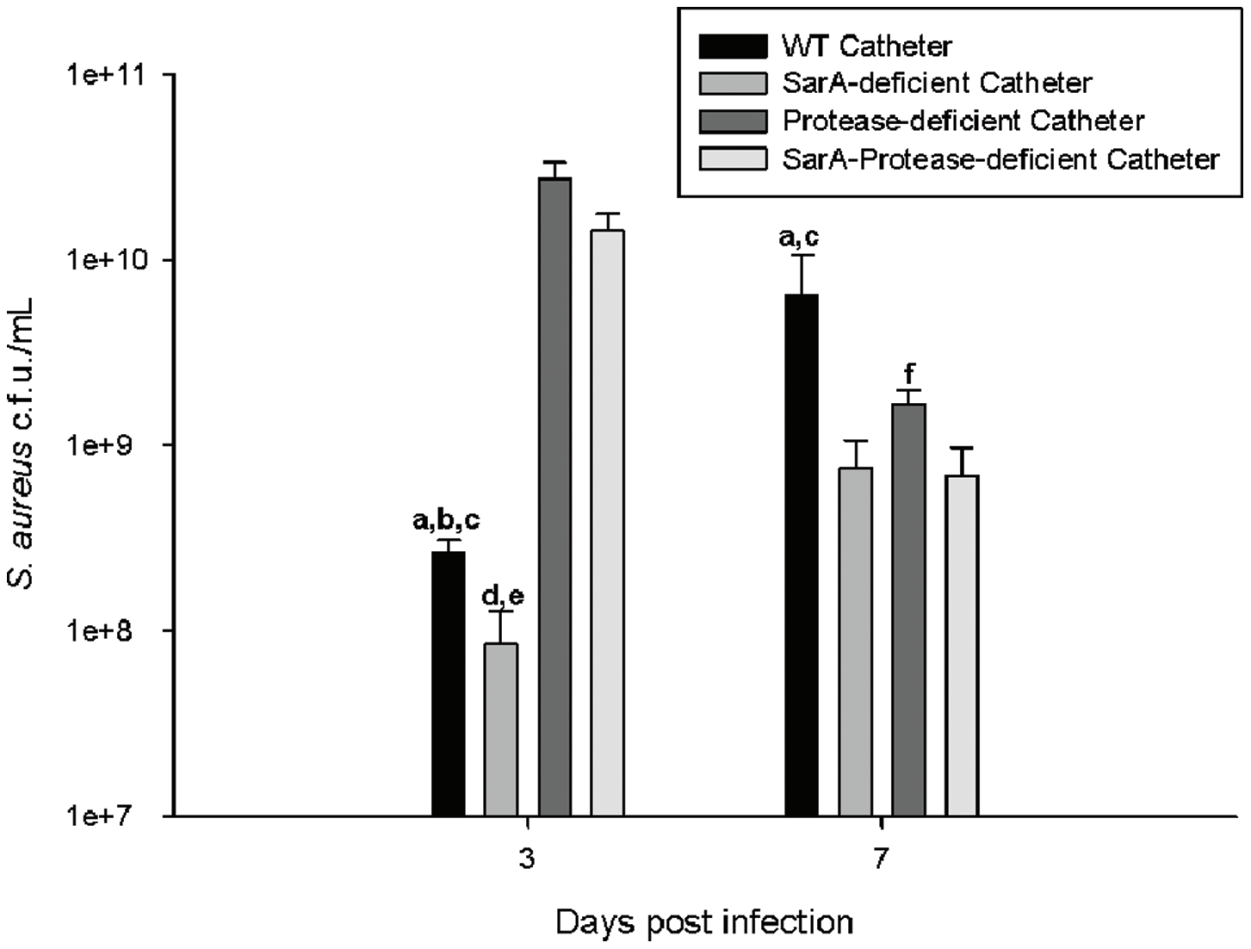
*sarA* and extracellular proteases significantly affect biofilm formation in a CNS catheter infection model. Infected catheters were removed, rinsed and sonicated for quantification of viable bacteria associated with wild type USA300 LAC (**WT catheter**), USA300 LACΔ*sarA* (**SarA-deficient catheter**), extracellular protease deficient USA300 LAC (**Protease-deficient catheter**), or SarA and extracellular protease deficient (**SarA-Protease-deficient catheter**) *S. aureus*. At all time points, bacterial burdens in brain parenchyma were 3–5 log lower than the corresponding catheter cultures (See Figure S4). **a** = p<0.05 WT vs SarA-deficient catheter; **b** = p<0.05 WT vs Protease-deficient catheter; **c** = p<0.05 WT vs SarA-Protease-deficient catheter; **d** = p<0.05 SarA-deficient vs Protease-deficient catheter; **e** = SarA-deficient vs SarA-Protease-deficient catheter; **f** = SarA-Protease-deficient vs Protease-deficient catheter (n = 13–15 mice/group/time point).

Mutation in the *sarA* regulatory locus has been shown to have broad effects on several staphylococcal virulence factors, including decreased accumulation of α-toxin, decreased PSMs and increased extracellular protease activity [13], [22], [25]. As noted above, the decrease in α-toxin accumulation cannot account for the heightened inflammatory profile observed *in vivo* during CNS infection. Similarly, as PSMs have also been demonstrated to be pro-inflammatory, decreased PSM production in staphylococcal *sarA* mutants should result in decreased inflammation rather than then increase observed in these studies [26], [27]. The third major virulence factor affected by *sarA* mutation in *S. aureus* are the extracellular proteases aureolysin (Aur), staphylococcal serine glutamyl endopeptidase A (SspA/V8) and its co-transcribed cysteine peptidase (SspB) and staphopain (ScpA), all of which are increased in the absence of sarA regulation [13], [18], [28]. To define the potential impact of altered protease production by the *sarA* mutant during CNS catheter infection, we compared the inflammatory profile of mice infected with a wild type USA300 LAC *S. aureus* strain with isogenic mutants deficient in *sarA* alone (SarA-deficient), the four major extracellular proteases described above (protease-deficient) and both *sarA* and the extracellular proteases (SarA-protease-deficient) [13]. This approach also allowed us to confirm the altered inflammatory phenotype observed with MSSA (ACH1719) versus MRSA (USA300 LAC) *S. aureus* isolates. At days 3 and 7 post-infection, all strains demonstrated significantly more bacteria associated with the catheter than the surrounding parenchyma, consistent with the above experiments with the MSSA strain ACH1719 (data not shown). Both the protease-deficient and *sarA*-protease deficient MRSA strains displayed greater biofilm formation, as evidenced by greater numbers of bacteria cultured from the catheters, at day 3 in congruence with prior reports of these mutants in peripheral infection models (Figure 7) [13]. This effect was transient and dissipated by day 7, suggesting that the impact of extracellular proteases is greatest during acute CNS catheter infection. Evaluation of CNS parenchymal titers revealed no dramatic differences between the WT or its isogenic mutants (Figure S4).

**Figure 8:**
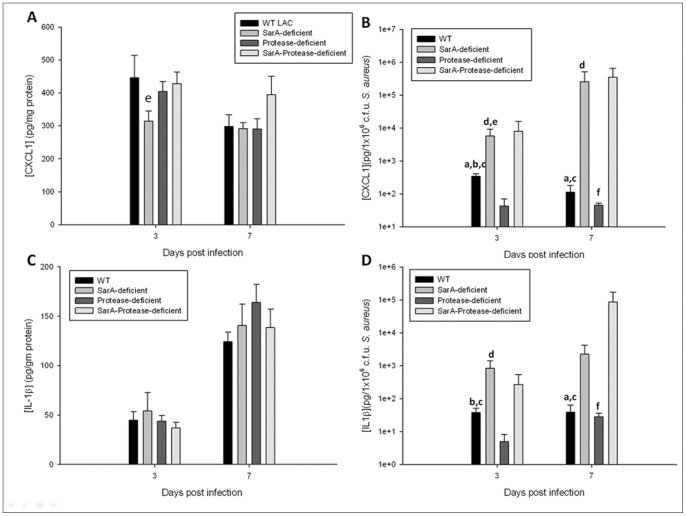
Inflammatory mediators are elevated in response to infection with *sarA* and *sarA-*protease mutants independently of protease activity. Catheter-associated infections were generated with wild type USA300 LAC (**WT catheter**), USA300 LACΔ*sarA* (**SarA-deficient catheter**), extracellular protease deficient USA300 LAC (**Protease-deficient catheter**), or SarA and extracellular protease deficient (**SarA-Protease-deficient catheter**) *S. aureus*. The tissue surrounding the infected catheters was homogenized and the resulting supernatant analyzed for levels of the pro-inflammatory mediators CXCL1 (A, B) and IL-1β (C, D). Results are presented as raw data (A and C) and after adjustment for divergent bacterial burdens (B and D). * = p<0.05 (n = 13–15 mice/group/time point).

To evaluate whether extracellular proteases, in conjunction with or independent of broader *sarA* effects, affect pro-inflammatory mediator production during CNS catheter infection, homogenates of catheter-associated tissues were analyzed for IL-1β and CXCL1 production by ELISA. When adjusted to account for divergent bacterial burdens, both mediators were significantly elevated in mice infected with the SarA*-* and SarA-protease-deficient MRSA strains in comparison with the wild type and protease-deficient MRSA strains (Figure 8). This relationship was also observed when measuring IL-17 levels (data not shown). This suggests that the heightened inflammation observed following CNS infection with a *sarA* mutant cannot be attributed to alterations in protease activity, given the increased inflammation observed in response to infection with the SarA-protease-deficient strain versus the protease-deficient strain.

## Discussion

Cerebrospinal fluid shunt infections are a frequent complication of hydrocephalus treatment, with significant associated costs in both medical expense and patient morbidity [2], [29]. These infections can cause shunt failure, increased seizure risk, decreased intellectual performance and increased mortality [2]. Because it is difficult to treat these catheter-associated biofilm infections in the brain, current guidelines recommend removal of the infected shunt, resulting in additional surgical procedures and extended hospital stays for affected children [4]. Thus, a better understanding of host-pathogen interactions is crucial to guide new treatment options for these CNS catheter infections.

Our recent studies using this murine model demonstrated a reproducible catheter-associated infection, with scanning electron microscopy findings consistent with biofilm formation [5]. In addition, increases in pro-inflammatory cytokines and chemokines were detected in the tissue surrounding infected catheters, as well as enhanced innate immune cell influx. These comparisons were made to responses elicited by sterile catheters, which allowed an important assessment of the injury/foreign body response. In the current study, we have defined these inflammatory changes in comparison to a planktonic infection (i.e. brain abscess), to better delineate the relative contribution of the biofilm itself to the CNS immune response. Here we report that catheter-associated biofilms are less inflammatory than planktonic infections, as evidenced by the fact that pro-inflammatory cytokine and chemokine expression was significantly reduced in animals with catheter infections caused by wild type *S. aureus* compared to brain abscesses induced by the same strain. This is consistent with the anti-inflammatory phenotype described in a peripheral model of *S. aureus* catheter-associated infection in the flank, in which the biofilm was found to induce lower levels of cytokines and chemokines than expected [7]. This diminished inflammatory response may play a role in the persistence of these infections, as effective immune clearance of the bacteria is impaired by the biofilm.

We next utilized a staphylococcal strain with a mutation in the regulator *sarA* as a tool to further explore the mechanisms responsible for this attenuated inflammatory response. A *S. aureus sarA* mutant was chosen as a representative-biofilm deficient strain because it has demonstrated decreased biofilm formation *in vivo* in multiple animal models of infection [10]–[12]. Using a SarA-deficient MSSA mutant (Δ*sarA*ACH1719), we observed a similar increase in inflammatory mediators during CNS catheter infection as was seen in the parenchymal brain abscesses, when compared with wild type catheter-associated infection. SarA is an important staphylococcal regulator, with broad effects, including control of several key virulence factors [10]–[12], [19], including decreased accumulation of α-toxin and PSMs in *sarA* mutants [13], [21], [22], [24]–[27], [30]. Based on the pro-inflammatory properties of both α-toxin and PSM, the decreased accumulation of these factors in *sarA* mutants should result in decreased inflammation rather than the increase observed in these studies. SarA-deficient staphylococcal strains have also been shown to have increases in the extracellular proteases Aur, SspA, and its co-transcribed SspB, and ScpA [13], [18], [28]. Alterations in extracellular proteases have been shown *in vitro* and *in vivo* to result in an increase in biofilm accumulation, as confirmed by the increased catheter-associated bacterial burdens in the protease-deficient and SarA-protease-deficient MRSA strains observed in the central nervous system in these experiments [13], [31]. However, little is known about the impact of increased protease production on the immune response to infection. By comparing levels of pro-inflammatory mediators in the CNS tissue surrounding the catheters infected with wild type, SarA-deficient, protease-deficient and SarA-protease-deficient MRSA strains, we were able to demonstrate that the increase in inflammation observed in infections with *sarA* mutants was independent of extracellular protease activity. Effects of *sarA* beyond regulation of these virulence determinants needs to be further explored to determine the specific mechanisms responsible for this attenuated CNS immune response.

Interactions between staphylococcal biofilms and the host immune response remain to be fully defined in either the CNS or the periphery. Both TLR2 and TLR9 ligands are known to be present in biofilms, yet the absence of neither receptor impacted biofilm growth in an *in vivo* model of *S. aureus* catheter-associated infection in the flank, suggesting there are redundant mechanisms available to assist the biofilm in evading the immune response [7]. Later studies in this model suggest that MyD88 signaling is needed for early containment and regulation of the macrophage response in biofilm infections, as lack of MyD88 signaling augmented polarization toward an anti-inflammatory M2 macrophage response [8]. Further demonstrating the complexity of host-pathogen interactions *in vivo*, a staphylococcal post-surgical joint infection model showed that IL-1β appeared to play a protective role in this setting through neutrophil recruitment, which also requires MyD88 for signaling via IL-1RI [32]. Other studies using a bone pin biofilm infection model have demonstrated a skewed immune response in which mice with a Th1-predominant phenotype are more easily infected and those with Th2-predominant phenotype easily clear infection, suggesting that the immune response to a biofilm is distinct from that of a parenchymal infection [6]. This has been demonstrated *in vitro* as well, with decreased macrophage phagocytosis of both *S. aureus* and *S. epidermidis* biofilms [7], [33]. Additionally, *in vitro* experiments with *S. epidermidis* found that macrophages were less pro-inflammatory when co-cultured with biofilm-capable versus biofilm-impaired strains of bacteria [33], similar to the results seen in our *in vivo* experiments comparing wild type *S. aureus* versus the biofilm-impaired *sarA* mutant strain of *S. aureus*. Collectively, these studies and our own work suggest that interactions between the host and staphylococcal biofilms are complex, with alterations in the immune response favoring bacterial persistence. Further work is needed to define the mechanisms responsible for this depressed immune response. To further explore the mechanisms of immune evasion in biofilm infection, we are currently adapting this CNS catheter infection model to generate infection with *S. epidermidis*, as we would expect the immune dampening to be more pronounced in infections with an organism with a more limited array of virulence factors [34]–[36].

Alterations in SarA have been extensively explored, as this presents a potential therapeutic target for treatment of staphylococcal disease given the important role of SarA in defining virulence. Several studies have demonstrated that infections caused by *sarA-*deficient strains of *S. aureus* are more susceptible to antimicrobial treatment compared to wild type strains, including subcutaneous catheter and endocarditis models [11], [20]. Given the enhanced inflammatory responses and bacterial clearance observed in our CNS catheter model following Δ*sarA* infection, it is reasonable to suspect that SarA inactivation also renders the biofilm more susceptible to immune clearance. This may further support the potential of SarA as a therapeutic target for the treatment of staphylococcal device infections. However, care must taken when evaluating this treatment in the CNS, given the increased mortality rate observed in response to Δ*sarA* in our experiments. This increased mortality may be mitigated by the addition of antibiotic therapy, given the increased response to antibiotics noted in other models testing *sarA-*deficient *S. aureus* strains; however, this needs to be carefully evaluated in the CNS [11], [20].

The decreased inflammatory response to biofilm infections in the CNS may explain the bacterial persistence and resultant difficulty in treating these infections in children. A better understanding of the complex interactions between the immune system and the biofilms that form along catheters in the brain could provide valuable information for screening patients at high risk for infections and for providing adjunctive therapy to current antibiotic treatment recommendations. The high costs to these children and their families in healthcare expenses and patient morbidity make new treatment and diagnostic modalities imperative for patients with CNS catheter infections.

## Materials and Methods

### Ethics Statement

This study was conducted in strict accordance with the recommendations in the Guide for the Care and Use of Laboratory Animals of the National Institutes of Health. The protocol was approved by the Institutional Animal Care and Use Committee of the University of Nebraska Medical Center (Approval IDs: 09-053-08-FC and 11-012-04-FC). All surgery was performed under ketamine/xylazine anesthesia, and every effort was made to minimize suffering.

### Mice

Male C57BL/6 mice (The Jackson Laboratory, Bar Harbor, ME; 8 weeks of age) were used for all studies. Each experiment was performed independently at least three times, with a minimum of 4–5 mice per experimental group at each time point.

### Bacterial Strains

Brain abscess and catheter-associated infections were generated using a MSSA strain obtained from a ventriculo-peritoneal shunt infection of a pediatric patient at Arkansas Children’s Hospital (ACH 1719), or its isogenic mutant missing the *sarA* regulator. This *sarA* mutant was kindly generated by the laboratory of Dr. Mark Smeltzer (University of Arkansas for Medical Sciences, Little Rock, AR) via φ-mediated transduction as previously described [37]. Additional catheter-associated infections were generated using USA300 LAC and its isogenic mutants lacking the *sarA* regulator, genes encoding the extracellular proteases (*aur, scpA, splABCDEF,* sspAB) or both *sarA* and the extracellular protease genes [13], [38]. For *in vitro* assays, UAMS-1 and USA300 LAC were also used for comparison as these are well-established biofilm-forming clinical isolates [7], [19], [39].

### CNS Catheter-associated Biofilm Infection

Silicone catheters were pre-coated with normal mouse serum and incubated with either 10^3^ cfu/ml ACH1719 WT or 2×10^4^ cfu/ml ACH1719 Δ*sarA* for 4 h to facilitate bacterial attachment (final bacterial burdens adherent to the catheter were 7×10^2^ cfu ±3×10^2^ and 3.5×10^3^ cfu ±8.7×10^3^ respectively). A larger initial bacterial burden in mice infected with ACH1719 Δ*sarA* was chosen given its decreased ability to form sustained biofilm infection as reported by others [11], [12], [19]. Catheters were rinsed and implanted intracerebrally into the lateral ventricle of mice using a stereotactic apparatus as previously described [5]. Briefly, mice were anesthetized with i.p. injections of ketamine and xylazine (100–200 mg/kg and 5–16 mg/kg respectively) and a 1 cm longitudinal incision was made in the scalp to expose the underlying skull sutures. A rodent stereotaxic apparatus equipped with a Cunningham mouse adaptor was used to implant *S. aureus-*coated catheters into the lateral ventricle using the following coordinates relative to bregma: +0.2 mm rostral, +1 mm lateral and −2 mm deep. A burr hole was made at these coordinates and the catheter gently inserted until it was level with the skull surface. The burr hole was sealed with bone wax to minimize bacterial efflux and bleeding. The skin incision was closed using surgical glue and the animals monitored over the post-operative course for clinical signs of infection or any post-operative complications. All animals survived the procedure, with mortality rates of 24% and 35% for the wild type and ACH1719 Δ*sarA* infected animals, respectively. Prior studies have demonstrated that animals implanted with sterile catheters survive the procedure and post-operative course indefinitely, with no mortality attributable to the catheter implantation [5]. Animals that survived long-term were sacrificed at the indicated intervals post-infection for evaluation of bacterial burdens and inflammatory indices. Data from any animal with negative bacterial cultures at the time of sacrifice, suggesting spontaneous resolution of infection or failure to establish infection, were excluded from further analysis.

### Generation of Experimental Brain Abscesses

Brain abscesses were induced by stereotactic injection of 10^4^ cfu *S. aureus* strain ACH1719 encapsulated in agarose beads into the striatum as previously described [14], [15]. The procedure was well-tolerated by all animals with a mortality rate <5% over the course of infection. All mice survived the procedure indefinitely and were sacrificed at the indicated time points to evaluate bacterial burdens and inflammatory indices. Data from any animal with negative bacterial cultures at the time of sacrifice, suggesting spontaneous resolution of infection or failure to establish infection, were excluded from further analysis.

### Generation of *S. aureus* Biofilms *in vitro*

To assess the ability of the ACH1719 *sarA* mutant to form a biofilm, overnight cultures of USA300 LAC, UAMS-1, MSSA ACH1719 and MSSA ACH1719 Δ*sarA* were incubated with TSB in a 96-well polystyrene microtiter plate pre-coated with mouse serum. After a 24 h incubation period and rinsing, the remaining biofilm was fixed with 100% EtOH and stained with 0.41% crystal violet for visualization [5], [19]. For confocal microscopy analysis, static biofilms were grown on sterile glass chamber slides (Fisher Scientific, Houston TX) treated with 20% human plasma (generous gift of Dr. Steve Carson, UNMC) in sterile carbonate-bicarbonate buffer overnight [40]. The plasma coating buffer was removed and each chamber inoculated with 2 ml ACH1719 or ACH1719 Δ*sarA* in TSB. Slides were incubated at 37°C under static aerobic conditions for 2 days and stained with Syto9 (1∶100 dilution in PBS; Invitrogen, Carlsbad CA) for visualization of the biofilm using a Zeiss laser scanning confocal microscope (Zeiss LSM 510, Oberkochen, Germany).

### Enumeration of Viable Bacteria from CNS Tissue and Catheters

Catheters and surrounding brain tissues were recovered from infected mice at days 3, 5, 7, 10 and 14 following infection. Brain tissues were homogenized for bacterial titer determination and inflammatory mediator measurement as previously described, allowing for quantitative culture and cytokine analysis from a single animal [41]. Briefly, catheters were removed and the surrounding brain tissue was sectioned within 1–2 mm on all sides. Catheters were removed from the brain tissue, rinsed in PBS to remove any non-adherent bacteria and sonicated in 500 µl of PBS. This solution was then serially diluted and cultured on tryptic soy agar (TSA) plates supplemented with 5% sheep blood as previously described [5], [10]. Brain tissues were homogenized in 500 µl sterile phosphate-buffered saline (PBS) supplemented with a complete protease inhibitor cocktail tablet (Roche, Basel Switzerland) and RNase inhibitor (Promega, Madison WI) using a Polytron homogenizer (Brinkmann Instruments, Westbury NY). A 20 µl aliquot was removed for culture via serial dilutions on agar plates as described above. The remaining slurry was centrifuged and the supernatant collected and stored at −80C until cytokine/chemokine analysis.

### ELISAs

Inflammatory mediators (IL-1β, IL-17, and CXCL1) were measured in brain tissue homogenates using commercially available ELISA kits according to the manufacturer’s instructions (R&D Systems, Minneapolis MN). Results were normalized to total bacterial titers of each tissue to correct for differences in bacterial burdens between wild type and mutant strains. Alpha toxin levels were measured using a custom-designed ELISA protocol. Briefly, wells of an ELISA microtiter plate were coated with purified α-toxin (Toxin Technologies, Sarasota FL) diluted in carbonate/bicarbonate buffer (pH 9.6) to generate a standard curve. The remaining wells were coated with the sample (100 µL per well) and incubated overnight at 4°C. The plates were washed and then blocked with 10%FBS/PBS for 2 h at room temperature (200 µL per well). After washing, wells were then incubated with a rabbit anti-α-toxin antibody (Sigma Aldrich, St. Louis MO) for 1 hour at room temperature (100 µL per well). The wells were again washed, whereupon 100 µL per well of an anti-rabbit IgG-HRP secondary Ab was added (Sigma Aldrich, St. Louis MO) for 1 h at room temperature. Following this incubation, the wells were washed, incubated for 15–20 min with TMB substrate in the dark until color development, and read using a plate reader at 450 nm Ex/575 nm Em.

### Quantitation of Immune Cell Infiltrates in Brain Tissues

Catheter-associated cells were recovered from the brain parenchyma for quantitation by FACS analysis as previously described [14], [15]. Briefly, mice were perfused to eliminate leukocytes from the vasculature and the entire infected hemisphere was removed to recover catheter-associated cells. Brain tissues were minced and gently filtered through a 70 µm nylon mesh strainer. The resulting slurry was subjected to collagenase/DNAse digestion to facilitate immune cell retrieval, and then centrifuged on a discontinuous Percoll gradient to remove myelin debris. Recovered cells were incubated in Fc Block™ (BD Biosciences, San Diego CA) to minimize non-specific antibody binding to Fc receptors and then stained with directly conjugated antibodies for FACS to detect neutrophils (Ly-6G^+^, CD11b^+^, CD45^hi^), macrophages (Ly-6G^−^, CD11b^+^, CD45^hi^), microglia (Ly-6G^−^, CD11b^+^, CD45^lo-intermediate^) and T lymphocytes (CD3^+^). All antibodies were purchased from BD Biosciences. Cells were analyzed using a BD FACSAria with compensation set based on the staining of each individual fluorochrome alone and correction for autofluorescence with unstained cells. Results were corrected based on catheter-associated bacterial titers to account for burden disparities between the ACH1719 wild type and ACH1719 Δ*sarA* strains.

### Statistics

Significant differences between experimental groups were determined using the unpaired Student *t-*test at the 95% confidence interval with Sigma Stat (SPSS Science, Chicago IL).

## Supporting Information

Figure S1 **ACH1719Δ** ***sarA*** **displays impaired biofilm formation** ***in vitro.*** The ability of ACH1719Δ*sarA*, wild type ACH1719, USA300 LAC, and UAMS-1 to establish a biofilm was determined using an *in vitro* microtiter plate assay with crystal violet staining (A) and confocal microscopy where bacteria were cultured on a glass chamber slide and stained with Syto9 (B).
(TIF)

Figure S2 ***sarA*** **exerts no significant effect on parenchymal spread of CNS infection.** The infected tissue was removed, homogenized and cultured to enumerate the bacterial burdens in the tissue surrounding the catheter infected with either wild type ACH1719 (**WT**) or ACH1719Δ*sarA* (**Δ** ***sarA***) *S. aureus*. Catheters were also removed, rinsed and sonicated for quantification of viable bacteria. **a** = p<0.05 WT brain vs catheter; **b** = p<0.05 Δ*sarA* brain vs catheter; **c** = p<0.05 WT catheter vs Δ*sarA* catheter; (n = 11–21 mice/group/time point).
(TIF)

Figure S3 **There is a trend toward increased peripheral immune cell influx during CNS catheter-associated infection in the absence of** ***sarA.*** Catheter associated cells were recovered from mice at days 3, 7 and 14 (day 14 not shown) post-infection, whereupon pooled tissue from 4–5 mice per group (wild type vs SarA-deficient) was analyzed for macrophage, microglia and T cell markers (A) as well as neutrophil markers (B), with representative histograms and isotype controls shown here (three independent replicates performed).
(TIF)

Figure S4 **There is no significant effect of extracellular proteases on parenchymal spread of catheter-associated infection.** Infected catheters were removed, rinsed and sonicated for quantification of viable bacteria associated with wild type USA300 LAC (**WT catheter**), USA300 LACΔ*sarA* (**SarA-deficient catheter**), extracellular protease deficient USA300 LAC (**Protease-deficient catheter**), or SarA and extracellular protease deficient (**SarA-Protease-deficient catheter**) *S. aureus*. The surrounding tissue was also homogenized for bacterial culture. At all time points, the bacterial burdens adherent to the catheters were 3–5 log higher than the corresponding brain parenchyma, although this difference was not statistically significant in the SarA-deficient catheters at either time point nor the SarA-Protease-deficient catheter at day 7 (p<0.05 for all other groups and time points). Bacterial burdens in the brain parenchyma were essentially equal, with the exception of a statistically significant different between the parenchymal bacterial titers in the wild type and protease-deficient catheters at day 7. **a** = p<0.05 WT parenchyma vs catheter; **b** = p<0.05 Protease-deficient parenchyma vs catheter; **c** = p<0.05 SarA-Protease-deficient parenchyma vs catheter; **d** = p<0.05 WT vs Protease-deficient parenchyma (n = 13–15 mice/group/time point).
(TIF)
