# Supplementary figures and images for: Staphylococcus aureus sarA Regulates Inflammation and Colonization during Central Nervous System Biofilm Formation
Source: PLoS One. 2013 Dec 30;8(12):e84089. doi: 10.1371/journal.pone.0084089 (PMC3875531; doi:10.1371/journal.pone.0084089)

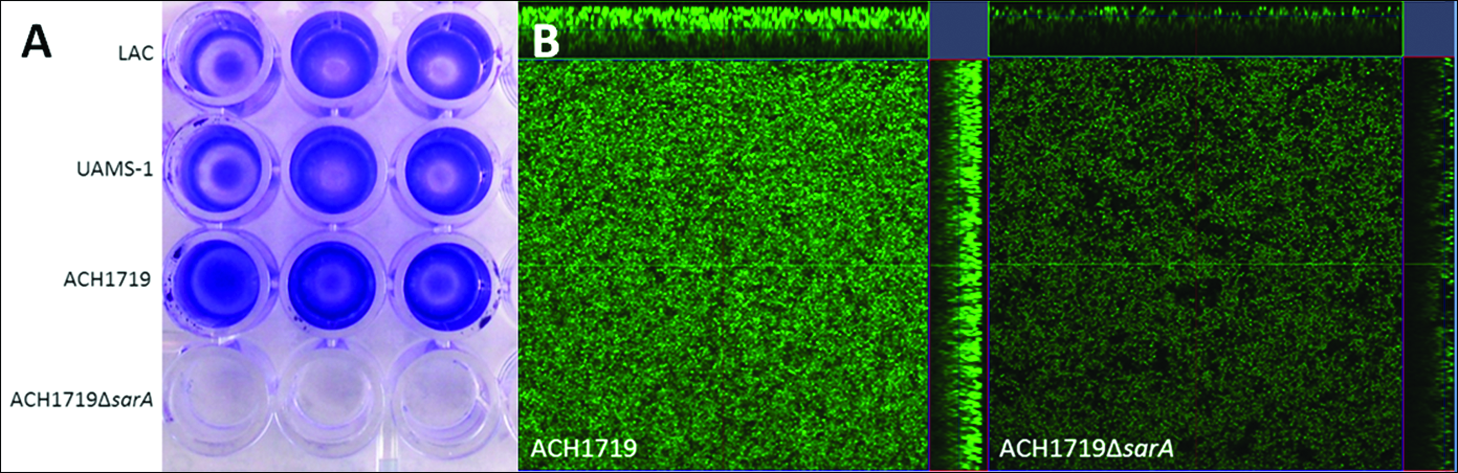

Supplement: Figure S1 — ACH1719Δ sarA displays impaired biofilm formation in vitro. The ability of ACH1719ΔsarA, wild type ACH1719, USA300 LAC, and UAMS-1 to establish a biofilm was determined using an in vitro microtiter plate assay with crystal violet staining (A) and confocal microscopy where bacteria were cultured on a glass chamber slide and stained with Syto9 (B). (TIF) [file pone.0084089.s001.tif]

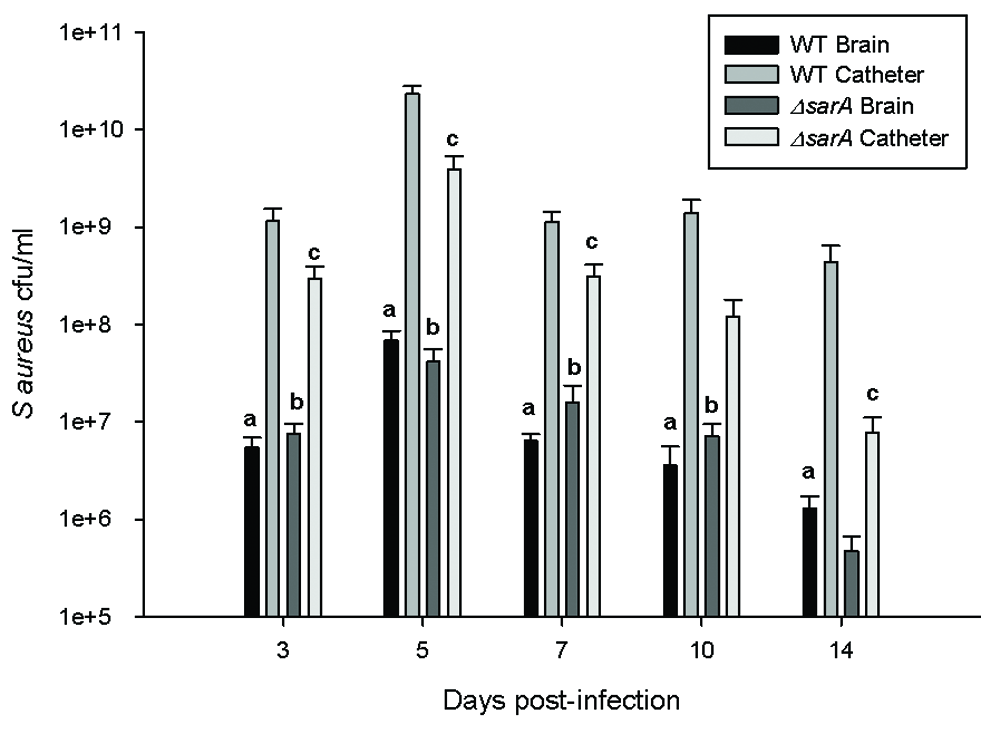

Supplement: Figure S2 — sarA exerts no significant effect on parenchymal spread of CNS infection. The infected tissue was removed, homogenized and cultured to enumerate the bacterial burdens in the tissue surrounding the catheter infected with either wild type ACH1719 (WT) or ACH1719ΔsarA (Δ sarA) S. aureus. Catheters were also removed, rinsed and sonicated for quantification of viable bacteria. a = p<0.05 WT brain vs catheter; b = p<0.05 ΔsarA brain vs catheter; c = p<0.05 WT catheter vs ΔsarA catheter; (n = 11–21 mice/group/time point). (TIF) [file pone.0084089.s002.tif]

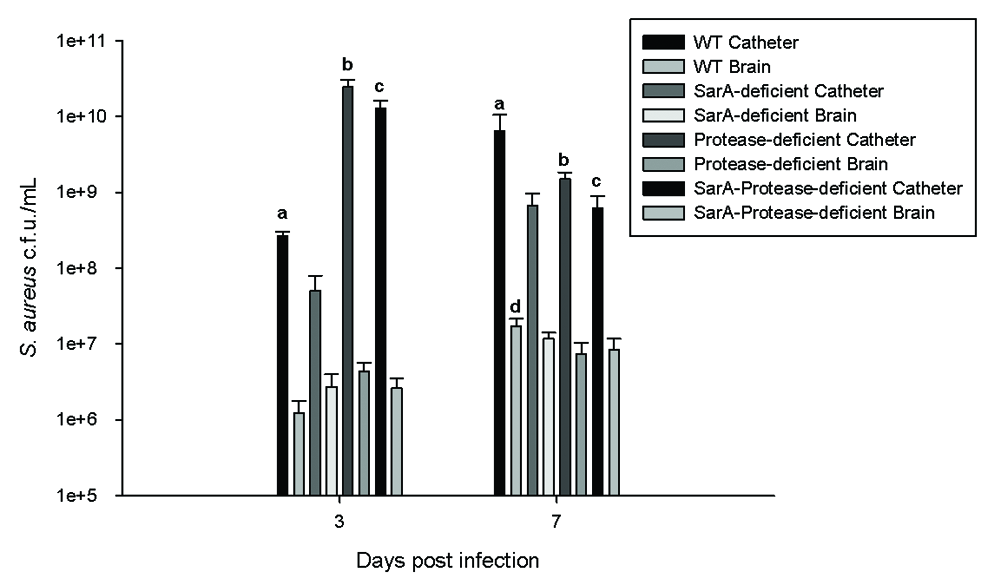

Supplement: Figure S4 — There is no significant effect of extracellular proteases on parenchymal spread of catheter-associated infection. Infected catheters were removed, rinsed and sonicated for quantification of viable bacteria associated with wild type USA300 LAC (WT catheter), USA300 LACΔsarA (SarA-deficient catheter), extracellular protease deficient USA300 LAC (Protease-deficient catheter), or SarA and extracellular protease deficient (SarA-Protease-deficient catheter) S. aureus. The surrounding tissue was also homogenized for bacterial culture. At all time points, the bacterial burdens adherent to the catheters were 3–5 log higher than the corresponding brain parenchyma, although this difference was not statistically significant in the SarA-deficient catheters at either time point nor the SarA-Protease-deficient catheter at day 7 (p<0.05 for all other groups and time points). Bacterial burdens in the brain parenchyma were essentially equal, with the exception of a statistically significant different between the parenchymal bacterial titers in the wild type and protease-deficient catheters at day 7. a = p<0.05 WT parenchyma vs catheter; b = p<0.05 Protease-deficient parenchyma vs catheter; c = p<0.05 SarA-Protease-deficient parenchyma vs catheter; d = p<0.05 WT vs Protease-deficient parenchyma (n = 13–15 mice/group/time point). (TIF) [file pone.0084089.s004.tif]
